# Exploring the expectations of patients with advanced cancer receiving palliative care services: a qualitative study in mainland China

**DOI:** 10.3389/fpubh.2026.1831825

**Published:** 2026-05-20

**Authors:** Jiamin Xu, Jiahuan Gu, Tintin Zhang, Lei Li, Lingchang Shan, Haiyan Zhu

**Affiliations:** 1 Department of Gastrointestinal Surgery, Shaoxing People's Hospital, The First Hospital of Shaoxing University, Shaoxing, Zhejiang, China; 2 Department of Intensive Care Unit, Shaoxing People's Hospital, The First Hospital of Shaoxing University, Shaoxing, Zhejiang, China; 3 Department of Medical Oncology, Shaoxing People's Hospital, The First Hospital of Shaoxing University, Shaoxing, Zhejiang, China; 4 School of Medicine, Shaoxing University, Shaoxing, Zhejiang, China; 5 Department of Nursing, Shaoxing People's Hospital, The First Hospital of Shaoxing University, Shaoxing, Zhejiang, China

**Keywords:** advanced cancer, expectations, palliative care, qualitative study, traditional Chinese medicine

## Abstract

**Purpose:**

To explore the expectations of patients with advanced cancer receiving palliative care services in mainland China, and to provide empirical evidence for constructing localized, culturally congruent palliative care service models integrated with Traditional Chinese Medicine (TCM) characteristics.

**Methods:**

A descriptive phenomenological qualitative design was adopted, and purposive sampling was used to select 11 patients with advanced cancer in the palliative care ward of a tertiary general hospital in Zhejiang Province for semi-structured in-depth interviews. Colaizzi’s seven-step analytical method was applied for thematic analysis, and multiple strategies were used to ensure the trustworthiness of the study.

**Results:**

Four main themes and 13 sub-themes regarding end-stage patients’ expectations of palliative care services were identified in this study: expectations for palliative care service settings; expectations for the competence of palliative care providers; expectations for palliative care-related support systems; and expectations for TCM-integrated palliative care services.

**Conclusion:**

Patients with advanced cancer have diverse and individualized expectations for palliative care services, with a prominent demand for TCM-integrated care. Healthcare providers and policymakers should develop localized palliative care service models based on patients’ expectations, improve the tripartite integrated care system of hospitals, communities and families, and integrate TCM concepts and techniques into palliative care to meet the end-of-life needs of patients under the Chinese sociocultural context.

## 1 Introduction

Palliative care was a patient-centered, comprehensive healthcare model. It not only improved the quality of life for patients at the end of life but also assisted families in coping with death rationally, while optimizing the allocation of healthcare system resources (1). Its advantages had been supported by global evidence-based medicine, making it a crucial direction for end-of-life care models (2).

Currently, China has already entered an aging society ahead of schedule, accompanied by a sharp increase in cancer patients. However, due to a lack of palliative care education and the influence of traditional Confucian “filial piety” values, many people mistakenly equate palliative care with “giving up treatment.” They believe it violates the principle of “doing everything possible” to save the lives of terminally ill patients (3). China’s palliative care development has been shaped by a complex interplay of systemic, cultural, and structural factors. In 2017, the Chinese government introduced a series of policies to promote palliative care, significantly advancing its development. Nevertheless, there remains a considerable gap compared to countries where palliative care was established earlier. Currently, China’s palliative care model exhibits several shortcomings: Insufficient service resources and a shortage of specialized professionals. Inadequate multidisciplinary collaboration mechanisms; Imperfect national policies and healthcare insurance systems. Therefore, government leadership is essential to drive the further development of palliative care in China (4).

Studies have explored palliative care services primarily from a hospital-centric perspective, failing to incorporate the preferences and expectations of patients with advanced cancer (5). Given China’s vast territory and diverse ethnic groups, customs and beliefs vary significantly, leading to differing expectations toward palliative care services. Therefore, this study adopted a descriptive phenomenological qualitative method to explore the expectations of patients with advanced cancer for palliative care services, with a focus on their demand for TCM-integrated care, aiming to provide a reference for constructing a localized palliative care service model adapted to China’s sociocultural context.

## 2 Methods

### 2.1 Study design

This study adopted a descriptive phenomenological qualitative design, which is rooted in Husserl’s phenomenological philosophy and focuses on exploring the lived experiences and subjective perceptions of research participants. This design is particularly suitable for exploring understudied phenomena such as advanced cancer patients’ expectations for palliative care, as it can avoid the interference of pre-set theoretical frameworks and fully capture the authentic feelings and needs of participants. The study was reported in strict accordance with the Standards for Reporting Qualitative Research (SRQR) guidelines (6).

### 2.2 Researcher characteristics and reflexivity

All semi-structured interviews were conducted by the first author, a female nursing postgraduate with systematic training in qualitative research methods (including descriptive phenomenology and Colaizzi’s analysis method) and more than 2 years of clinical experience in cancer nursing and palliative care. The researcher maintained a reflective journal throughout the research process, recording personal preconceptions, emotional responses and interaction details with participants in a timely manner. For example, the researcher initially held the preconception that “older patients prefer home care”, and this bias was continuously examined and corrected in the interview and data analysis process. The reflective journal was used to monitor the impact of the researcher’s subjectivity on the research process, ensuring the methodological rigor of the study. The specific roles of each research team member: Xu Jiamin collected all data; Xu Jiamin and Shan Lingchang completed and cross-checked audio transcription; Xu Jiamin, Zhu Haiyan and Zhang Tintin conducted thematic analysis (initial coding, parallel coding, theme refinement respectively); Gu Jiahuan optimized the interview protocol and Li Lei collated literature and did quality control.

### 2.3 Setting and sampling

Purposive sampling combined with the maximum variation sampling principle was used to select patients with advanced cancer receiving palliative care in the Department of Medical Oncology of a tertiary general hospital in Zhejiang Province from January 2025 to May 2025. Eligible participants were referred by attending physicians and nurses, and voluntarily signed the informed consent form after being fully informed of the research purpose, procedures, rights and obligations.

The inclusion criteria were as follows: (1) Age≥18 years old; (2) Histopathologically confirmed as malignant neoplasm; (3) Patients entering palliative care phase; (4) Patients formally recognized as having exhausted disease-modifying therapies; (5) Organized thought content with intact comprehension and verbal expression. The exclusion criteria included: (1) cognitive impairment; (2) unstable condition or refusal to participate.

### 2.4 Ethical considerations

This study was approved by the Medical Ethics Committee of Shaoxing People’s Hospital (Ethics number: 2024-Scientific research project no. 033-Y-01). All study procedures complied with the Declaration of Helsinki and local ethical regulations. All researchers strictly adhered to the principle of informed consent. Prior to interviews, participants received written information about the research aims and procedures, and signed consent forms confirming their voluntary participation. All data were anonymized by replacing names with codes (e.g., P1, P2). To minimize psychological risks, interviewees were reminded of their right to skip questions or withdraw at any time. The researcher acknowledged potential power imbalances and maintained reflexivity throughout the process. No member outside the research team had access to raw data or unredacted materials.

### 2.5 Data collection

This study was guided by descriptive phenomenology within qualitative research, employing face-to-face, semi-structured in-depth interviews. Based on the research objectives, the interview protocol was developed through literature review, expert consultation, and pre-interviews with two advanced cancer patients (7). The interview protocol included the following questions: ① How much do you know about palliative care? ② Do you feel that palliative care services are helpful to you? ③ What do you think about the current state of palliative care services? ④ What would your ideal palliative care services look like? ⑤ What kind of help and support do you need the most?

Before the interviews, the researcher thoroughly explained the study’s purpose and methods to the participants, informed them that the interviews would be audio-recorded, and scheduled the interview time and location after obtaining their consent. All face-to-face semi-structured in-depth interviews were conducted in the quiet single wards of the tertiary general hospital. The interviews were conducted following the principles of voluntariness and confidentiality. Given the generally weak physical condition of patients with advanced cancer, each interview was limited to within 30 min to minimize any potential adverse effects on the patients and to ensure interview quality. During the interviews, no leading questions were asked, and no interventions were made. Key content and non-verbal data were documented in a timely manner, with careful attention paid to interviewing techniques.

In terms of privacy protection, we adopted multi-dimensional strict measures to ensure the confidentiality of the interview process and content: first, only the interviewer (the first author) and the participant were present during each interview, and all medical staff and family members were requested to temporarily leave the ward; second, the interview area was separated by a curtain to further avoid visual disturbance; third, the audio recording equipment was placed in a visible position of the participant with explicit informed consent, and the recording files were immediately labeled, encrypted and stored after the interview; fourth, all personal identifying information of the participants was anonymized with codes (P1-P11) in the subsequent transcription and data analysis process, and the original interview records were only accessible to the core research team.

### 2.6 Data analysis and quality control

#### 2.6.1 Data analysis

Within 24 h after each interview, the audio recordings were verbatim transcribed into text, and the on-site notes and non-verbal behaviors were integrated into the transcripts to form the original data. Colaizzi’s seven-step analytical method (8) was strictly applied for thematic analysis, and the specific steps were as follows:

- Familiarization with data: The research team repeatedly read all transcripts to fully understand the original data and record initial impressions and insights.
- Extraction of significant statements: Extract sentences/paragraphs that are closely related to the research theme (patients’ expectations for palliative care).
- Formulation of meanings: Encode and interpret the significant statements to form the initial meaning units.
- Clustering of meanings: Classify and merge the initial meaning units with similar connotations to form sub-themes.
- Development of themes: Summarize and refine the sub-themes to form the main themes that can reflect the core content of the research.
- Validation of themes: Compare the formed themes with the original data to ensure that the themes are consistent with the participants’ expressions and can fully reflect their expectations.
- Production of the final report: Construct the research results based on the themes, and combine the participants’ verbatim quotes to present the results authentically.

Example of the analytical process:

*Significant statement (P5)*: “After each morphine injection, I feel extremely drowsy and don’t even have the energy to talk with my family. If acupuncture, massage, or other external therapies could help reduce my pain and keep my mind clearer, that would be ideal.”

*Formulation of meaning*: Dissatisfaction with the side effects of Western medicine analgesics; expectation of TCM external therapies for pain relief; hope to maintain a clear mind for family communication.

*Clustering of meaning*: Pursuit of natural and gentle treatment approaches in TCM-integrated palliative care.

Development of theme: Expectations for TCM-integrated palliative care services.

#### 2.6.2 Quality control

To ensure the trustworthiness of the qualitative research results, the research team adopted multiple strategies corresponding to the four core criteria of qualitative rigor: credibility, dependability, confirmability and transferability.

*Credibility*: (1) Member checking: After the initial theme analysis was completed, the research results were fed back to 5 participants with different characteristics (age, education level, disease type), and their opinions and suggestions were collected to revise and perfect the themes to ensure that the results were consistent with the participants’ subjective experiences; (2) Prolonged engagement: The first author had continuous clinical contact with the participants before and after the interview, which enhanced the mutual trust between the researcher and the participants; (3) Triangulation: Two researchers independently coded the original data, and the third researcher mediated the differences in coding until a consensus was reached, ensuring the objectivity of the coding and theme extraction.

*Dependability*: (1) Peer debriefing: The research team regularly held debriefing meetings with 2 qualitative research experts who were not involved in the data collection, and reported the research process, coding results and theme formation to the experts for comments and guidance; (2) Detailed audit trail: A complete research record was established, including the interview outline, informed consent form, audio recordings, transcripts, coding notes, reflective journals and expert comments, so that the research process could be traced and repeated.

*Confirmability*: The research team strictly abided by the principle of “data-driven” in the data analysis process, avoided imposing pre-set theoretical frameworks on the original data, and the final themes were all extracted from the participants’ authentic expressions. The encrypted storage of research data and the independent coding of multiple researchers also avoided the researcher’s subjective bias affecting the research results.

*Transferability*: The study provided detailed descriptions of the research setting, sampling process, participant characteristics (Table 1) and research methods, and presented the research results combined with verbatim quotes of the participants. Readers can judge the applicability of the research results to other contexts (e.g., other regions, other types of cancer patients) according to the above information.

**Table 1:** The general characteristics of the respondents (*n* = 11).

No.	Gender	Age	Educational level	Occupation	Marital status	Religious belief	Disease types	Primary caregivers
N1	Female	54	Junior college	Bank staff	Married	No	Gastric cancer	Spouse
N2	Male	48	Undergraduate course	Technician	Married	No	Lung cancer	Spouse
N3	Male	65	Primary school	Retired worker	Married	Christian	Lung cancer	Spouse
N4	Male	52	Middle school	Freelancer	Married	No	Gastric cancer	Caregiver
N5	Female	65	Middle school	Retired teacher	Married	No	Colorectal cancer	Caregiver
N6	Female	32	Undergraduate course	Technician	Single	No	Breast cancer	Parent
N7	Male	68	Primary school	Farmer	Married	No	Prostate cancer	Spouse
N8	Male	47	Master’s Graduate	Civil servant	Married	No	Liver cancer	Spouse
N9	Male	76	Senior high school	Retired official	Married	No	Lung cancer	Caregiver
N10	Female	63	Primary school	Farmer	Married	No	Ovarian cancer	Spouse
N11	Male	72	Middle school	Retired worker	Widowed	No	Lung cancer	Caregiver

## 3 Results

**Figure 1:**
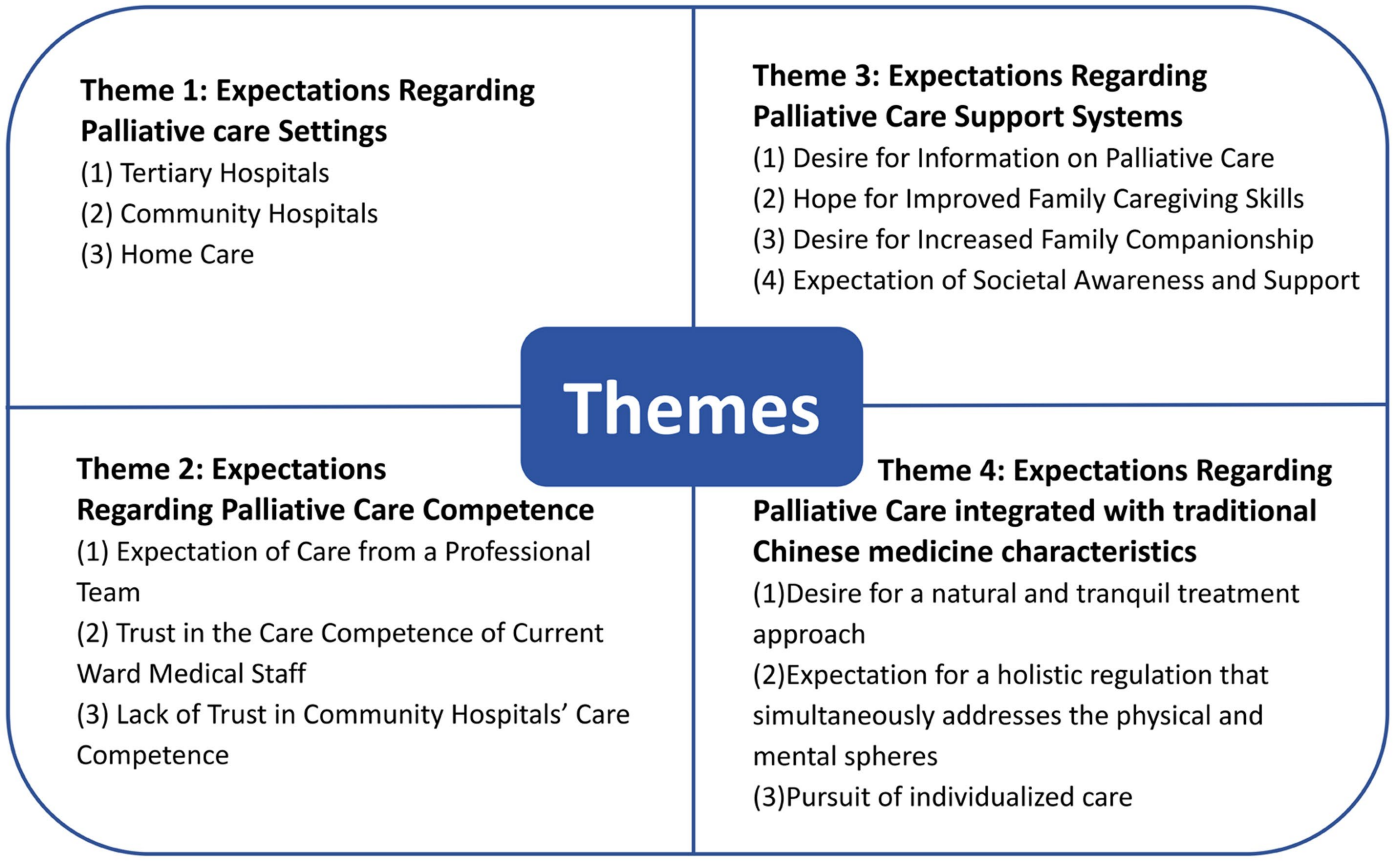
Four main themes and 13 sub-themes regarding end-stage patients’ expectations of palliative care services.

A total of 11 participants were included, whose characteristics were summarized in Table 1. As shown in Figure 1, four main themes and 13 sub-themes regarding end-stage patients’ expectations of palliative care services were identified in this study.

### 3.1 Theme 1: expectations for palliative care service settings

All participants expected a comfortable and reassuring palliative care environment, and their choices of care settings showed a diversified pattern with tertiary hospitals as the primary choice, and community hospitals and home care as supplements, which was a trade-off between medical safety and quality of life.

1 Tertiary hospitals.

Tertiary hospitals, known for their abundant medical resources and extensive expertise, were the preferred choice for most patients in this study.

N1: “Tertiary hospitals have comprehensive equipment and advanced skills. I feel truly at ease staying here.”

N5: “The service in large hospitals is considerate. When I have trouble sleeping at night, besides medication, the nurses offer aromatherapy lamps and play music for me.” (frequent nodding).

N8: “Tertiary hospitals have diverse palliative care teams. I trust they can help me pass away with dignity.”

N9: “I’ve been living with this illness for seven years and have been to many hospitals of different sizes. After comparing, I still prefer large hospitals.”

2 Community hospitals.

Community hospitals offer advantages such as convenience, a tranquil environment, and proximity to home. Three patients expressed willingness to receive care there.

N3: “There’s a church next to the community hospital, making it easy for my fellow believers to visit, talk, and pray with me.”

N10: “Community hospitals aren’t overcrowded, so it’s quiet and peaceful—just the environment I prefer.”

N7: “Nowadays, doctors and nurses from large hospitals regularly visit community hospitals, so I’m not worried about the quality of care.”

3 Home care.

Only two patients preferred receiving palliative care at home, while others indicated they would choose home care only when very close to the end of life.

N2: “I’ve always been busy with work and spent little time at home. Since being in the hospital doesn’t make much difference now, I’d rather be at home with my parents, wife, and children. That’s where I most want to be.”

N6: “I’ve been to the hospital too often over the past two years. The sight of white walls makes me anxious and suffocated. In the final phase of my life, I hope to spend it comfortably in my own familiar home.”

N4: “According to our customs, we must return home at the very end—but only when I’m no longer lucid.”

### 3.2 Theme 2: expectations for the competence of palliative care providers

As life approaches its end, patients desire high-quality care.

1 Expectation of care from a professional team.

Advanced cancer patients often experience multifaceted issues—physical, psychological, and spiritual—requiring collaborative support from a multidisciplinary professional team.

N2: “Lately, I’ve been in pain all over, especially at night. I’ve been taking painkillers for three days, but they aren’t working well. Could a pain management specialist come see me?” (frowning).

N4: “I used to struggle with why I got cancer and felt deeply depressed. Dr. Ye (the psychologist) is different—after two sessions with her, things suddenly made sense to me. I’d like to see her again.”

N6: “My parents often restrict what I can eat, but after your nutritionist visited last week, I finally got to eat what I wanted.” (smiling) “I hope they can come regularly.”

2 Trust in the care competence of current ward medical staff.

Due to repeated hospitalizations, advanced cancer patients have built trusting and dependent relationships with the medical staff and prefer to continue receiving care from them.

N1: “I’ve always been hospitalized in the oncology department. The doctors and nurses here understand my condition and temperament well—they know what I need without me having to explain.”

N5: “The medical staff here have been taking care of me for months, and they’re very thorough.”

3 Lack of trust in community hospitals’ care competence.

Patients often doubt community hospitals’ specialized technical capabilities or find them lacking essential medical resources.

N11: “The problem with community hospitals is their inadequate skills. I have a pressure sore on my hip, and after several dressing changes there, it actually got worse.”

N7: “Last time my home pain medication ran out, I went to the nearby community hospital, but they said they didn’t carry it.”

### 3.3 Theme 3: expectations for palliative care-related support systems

1 Desire for information on palliative care.

Most advanced cancer patients expressed a need to understand their condition, available palliative care methods, and relevant healthcare reimbursement policies.

N4: “I don’t know how long I have left. There are many matters I need to settle with my company and family, but I don’t want to discuss arrangements too early when faced with my grieving parents and wife. Please keep me informed about any changes in my condition so I can make arrangements in time.”

N8: “I’ve heard of palliative care, but I’d like to learn more about the specific methods involved.”

N5: “Are there special charges for palliative care services? Is it covered by health insurance?”

2 Hope for improved family caregiving skills.

Advanced cancer patients are not only physically frail but may also require professional care for issues like pressure sores, feeding difficulties, or tube management, areas where family members often lack experience and skills.

N1: “My husband has never been good at caregiving. Now, whenever the nurses come, I make sure he watches and learns carefully.”

N2: “I have a tube attached, and the nurses usually handle the care. If I go home, my mother will be responsible for me. Could the nurses teach her what to do?”

3 Desire for increased family companionship.

Receiving support and companionship from family and friends, and fostering warm, intimate, and caring relationships, are vital components of palliative care.

N11: “My son works in another city, and my spouse passed away early. He only came back when I was first hospitalized. It’s been eight months, and he hasn’t returned since. Sometimes I really miss him.” (gazing out the window).

N6: “Lately, I often think of old friends and past moments. I wonder if I’ll ever have the chance to gather with them again.” (with sadness).

4 Expectation of societal awareness and support.

Only when society collectively engages in and supports palliative care can advanced cancer patients experience dignity and peace in their final journey.

N2: “palliative care is still unfamiliar to many. When a friend visited recently and learned I was receiving palliative care, they looked shocked, mistaking it for euthanasia. They had no idea this type of care existed. There’s far too little public awareness.”

N7: “Over the years, I’ve spent a lot on medical treatments, and my son hasn’t been able to earn much. It’s worrying. I don’t know if there are any social subsidies for someone in my situation?” (sighing).

### 3.4 Theme 4: expectations for TCM-integrated palliative care services

This study found that there is a profound expectation among advanced cancer patients for the integration of TCM concepts and techniques into palliative care services. Such expectation does not aim to replace modern medicine, but rather serves as an important supplement and empowerment, seeking to enhance the quality of end-of-life experience in a gentler, more holistic, and culturally congruent manner.

1 Desire for a natural and tranquil treatment approach.

Most patients expressed a preference for natural therapies with minimal invasiveness and fewer side effects. They voiced concerns about the potential for addiction, drowsiness, and gastrointestinal reactions associated with long-term use of Western medications (such as opioid analgesics) and hoped that Traditional Chinese Medicine could offer a gentler alternative or complementary approach.

N12: “I’ve already taken so many medications. I’ve heard that TCM offers a gentler approach to recuperation, one that fundamentally reinforces the body’s vital energy. I’d like to try it, hoping to feel better without adding more chemical drugs.”

N5: “After each morphine injection, I feel extremely drowsy and don’t even have the energy to talk with my family. If acupuncture, massage, or other external therapies could help reduce my pain and keep my mind clearer, that would be ideal.”

N9: “I saw the patient in the next bed receiving moxibustion. The room was filled with a pleasant herbal aroma, and he said he felt a warm, relaxing sensation afterwards. I’m very interested in trying it myself. I’ve always felt that such a practice, passed down for thousands of years, must have its benefits.”

2 Expectation for a holistic regulation that simultaneously addresses the physical and mental spheres.

The patients were concerned not only with the alleviation of physical symptoms but also with attaining mental and emotional tranquility. They intuitively resonated with the TCM concept of the unity of body and spirit, expressing hope that TCM interventions could address both their physical suffering and psychological distress.

N4: “For my condition (referring to the tumor), Western medicine says it’s a physical growth, but Traditional Chinese Medicine often speaks of illnesses originating from the heart. I hope my doctors can not only review my lab reports but also help me resolve this internal emotional stagnation.”

N8: “The psychologist’s counseling helps me understand things rationally, but I still feel an inner restlessness. I’ve heard that practicing Tai Chi or doing breathing exercises can help calm the mind. I really want to learn, hoping to find some peace of mind during this final phase of my life.”

N11: “When the nurse massaged my head and shoulders, I felt not only physical relaxation but also as if the emotional congestion inside me was released. I particularly felt the urge to have a good cry and then sleep soundly. I wish we could have more of this kind of care.”

3 Pursuit of individualized care.

Many patients recognized that their symptoms and constitutions differed from others, leading them to perceive standardized Western medical treatments as insufficient. They expressed hope that the TCM approach of syndrome differentiation and treatment could provide them with personalized care plans.

N2: “The three of us in the ward all complain of pain, yet the painkillers prescribed are largely the same. But besides the pain, I also feel extremely fatigued, experience spontaneous sweating, and have a poor appetite. I hope TCM can tailor a regimen specifically for my individual condition-such as specific dietary adjustments and herbal formulas—based on these particular symptoms.”

N7: “I asked my doctor about my constitution type-whether it is cold or hot, deficient in yin or yang? Understanding this would give me clear guidance on what foods to choose in my daily life.”

## 4 Discussion

This study adopted a descriptive phenomenological method to explore the expectations of 11 patients with advanced cancer for palliative care services in mainland China, and identified four core themes, with the TCM-integrated palliative care service as a prominent and culturally specific expectation. The findings not only reflect the patient-centered core of palliative care, but also reveal the unique needs of Chinese advanced cancer patients under the influence of sociocultural factors, which provides empirical evidence for the construction of a localized palliative care service model in China.

This study found that patients’ expectations for palliative care settings presented a diversified pattern, dominated by tertiary hospitals and supplemented by community hospitals and home-based care. Behind this choice was a complex trade-off made by patients between medical safety and quality of life. The overwhelming preference for tertiary hospitals was primarily based on deep trust in their abundant medical resources, advanced technology, and professional teams. Well-equipped facilities and superior technical competence were the core sources of patients’ sense of security. This was closely related to the long-standing reliance of the Chinese public on large public hospitals (9, 10) and the fear of sudden symptoms (such as pain or dyspnea) among end-of-life patients. Patients regarded tertiary hospitals as the most reliable refuge in the final stage of life. However, this also raised a critical question for China’s palliative care service system: how to effectively integrate the philosophy and practice of palliative care into tertiary hospitals to avoid excessive medical intervention. Specific measures can be taken to integrate palliative care into tertiary hospitals and reduce unnecessary medical interventions: dedicated palliative care wards or subspecialties can be established, with clinical pathways focused on symptom control, comfort care, and improved quality of life to avoid redundant tests and treatments (11); training on palliative care can be strengthened for medical staff to foster the concept of early palliative and full-cycle care (12); multidisciplinary teams can be formed to conduct joint assessments and provide individualized interventions; performance appraisal systems can be optimized by incorporating quality of life, symptom relief, and patient satisfaction as key indicators (13); and two-way referral mechanisms with community and home-based care can be improved to achieve appropriate patient triage. In this way, active treatment and appropriate care can be balanced within a cure-oriented model, thereby reducing unnecessary medical interventions. In contrast, patients who opted for community hospitals or home-based care placed greater value on the familiarity of the environment and the proximity to family and community. The convenience of community hospitals and the emotional comfort of home care aligned well with the goal of enhancing the quality of life, as advocated by palliative care. However, these options were selected by only a very small number of patients, with the fundamental barrier being the lack of professional competence in primary medical institutions and home-based care. As demonstrated by the experiences of N11 and N7, the insufficient capability of community hospitals in managing specialized symptoms (such as pressure ulcer care and the supply of strong opioids) directly undermined patients’ confidence in choosing these options. Therefore, constructing a tripartite integrated care model linking hospitals, communities, and families is key for future development (14). Only when tertiary hospitals can act as technical supports and referral backbones, empowering community and home care through mechanisms such as medical alliances, can patient concerns be genuinely alleviated, enabling rational triage and distribution across care settings.

Patients’ expectations regarding service capabilities profoundly reflected the palliative care philosophy of “whole person, whole family, whole process, and whole team.” First, patients explicitly expressed a desire for a multidisciplinary professional team rather than a single healthcare provider. Their calls for specialized roles such as pain management physicians, psychologists, and nutritionists indicated that their needs had expanded beyond mere disease management to encompass the alleviation of holistic suffering, including pain, psychological distress, and nutritional imbalances. This aligns closely with the internationally recognized multidisciplinary team model in palliative care (15–17). Second, a noteworthy finding of this study was the deep trust and reliance patients placed on the medical staff in their current inpatient wards. This trust and familiarity were built through long-term patient-provider interactions. This insight suggests that when promoting palliative care, it should not be treated as a separate or isolated specialty. Instead, emphasis should be placed on integrating fundamental palliative care principles and skills into existing relevant departments, such as oncology and geriatrics, thereby fostering embedded palliative care capabilities. This approach not only enables a swift response to patient needs but also better aligns with the current realities of the healthcare system in China.

Patients’ expectations extended far beyond the scope of medical care, reaching into a broader support system. Informational support emerged as a primary need. Patients’ desire for knowledge regarding their disease progression, the specific content of palliative care, and relevant health insurance policies stemmed from their ultimate pursuit of autonomy over their lives (18, 19). They hoped to possess sufficient information while still mentally clear, enabling them to arrange their affairs and make informed decisions. This necessitates that healthcare providers move beyond traditional paternalistic approaches and engage in shared decision-making with patients and their families through more open and skillful communication. Finally, patients called for broader societal support (20). The prevalence of public misconceptions about palliative care—such as conflating it with euthanasia—coupled with pressing needs for financial assistance, highlights the current inadequacies in societal awareness and policy support for palliative care in China (21). Promoting public education on palliative care and integrating it into more comprehensive healthcare coverage and social assistance systems are fundamental measures to ensure equitable access to services and to guarantee that every end-of-life patient can depart with dignity.

This study revealed a profound expectation among end-of-life patients for the integration of Traditional Chinese Medicine (TCM) philosophies and techniques into hospice and palliative care. This finding not only enriched the understanding of patient needs but also provided a crucial foundation for developing a palliative care model with Chinese cultural characteristics. First, patients’ desire for a natural and gentle approach to care profoundly reflected their critical reflection on medicalized end-of-life experiences. While potent modern medical interventions, such as opioids, could alleviate symptoms, they often carried side effects like drowsiness and cognitive impairment, thereby depriving patients of precious lucid moments for emotional connection with their families (22). Patients sought TCM modalities, including herbal medicine, acupuncture, and moxibustion, in the hope of striking a balance between symptom control and maintaining mental clarity and personal integrity. This suggested that ideal palliative care should not merely manage symptoms but, more importantly, safeguard the patient’s personhood as a whole (23). The TCM philosophical principle of following the laws of nature offered a potential pathway to address the dilemmas posed by modern technological medicine. Second, patients’ expectation for simultaneous physical and psychological care highly aligned with the TCM holistic concept of body-spirit unity and the hospice principle of whole-person care (24). Patients were clearly aware of the intrinsic connection between physical suffering and psychological distress (as exemplified by N4’s description of “stagnant qi”). They were dissatisfied with service models that separated psychological counseling from somatic treatment, instead yearning for integrated interventions that could synchronously harmonize body and mind. For instance, practices such as Tai Chi and Baduanjin, along with Tui Na massage, were perceived as methods that could improve physical function while also calming the mind through breath and mental regulation (23). This expectation strongly called for the inclusion of professionals skilled in TCM emotional regulation theory and techniques within the palliative care team, or for training existing team members, to provide a more immersive service that seamlessly embeds psychosocial-spiritual care into daily physical care. Furthermore, patients’ pursuit of individualized care pointed directly to the limitations of standardized modern medical protocols and highlighted the core value of TCM’s treatment based on syndrome differentiation (25). End-of-life patients experienced complex and unique symptom clusters; as described by N2, even pain manifested with different concomitant symptoms (such as night sweats, fatigue, and poor appetite) across individuals. Standardized protocols were inadequate in responding to these vast inter-individual differences. Patients expressed a desire to understand their own constitution (e.g., cold/heat, deficiency/excess) and to receive tailored advice on diet, herbal medicine, and lifestyle. Integrating the TCM system of syndrome differentiation and treatment into palliative care assessment and practice could significantly enhance the precision of care and patients’ sense of control (26).

The international experience of characteristic medical systems in palliative care provides important enlightenment for the development of TCM-integrated palliative care in China: First, learn from Japan’s Kampo medicine and accelerate the standardization and institutionalization of TCM in palliative care, formulate unified clinical guidelines and efficacy evaluation standards, and realize the standardized production and application of TCM herbal medicines (27). Second, refer to India’s Ayurveda development model and strengthen the construction of community and home-based TCM palliative care services, train community medical staff and family caregivers in basic TCM palliative care skills, and expand the coverage of TCM palliative care services (28). Third, strengthen international cooperation and research on TCM palliative care, carry out joint research with foreign institutions on the efficacy and mechanism of TCM therapies in palliative care, publish high-quality international papers, and improve the international recognition of TCM-integrated palliative care (29). Fourth, combine the advantages of TCM with the characteristics of Chinese sociocultural context, take the “unity of body and spirit” and “syndrome differentiation and treatment” as the core, and build a TCM-integrated palliative care service model with Chinese characteristics that is suitable for the national conditions and in line with international palliative care concepts (30).

### 4.1 Limitations

This study has several limitations that need to be explicitly acknowledged:

*Small sample size and single-center design*: The study only included 11 participants from a tertiary general hospital in Zhejiang Province, and the sample size is relatively small. The research results may be affected by the regional medical level and sociocultural characteristics, and the generalizability is limited.

*Potential interviewer bias*: Although the researcher adopted a reflective journal and peer debriefing to reduce subjective bias, the first author’s identity as a clinical nurse may still affect the participants’ authentic expression of their feelings and needs.

*Lack of inclusion of other stakeholders*: The study only explored the expectations of advanced cancer patients themselves, and did not include the perspectives of family caregivers, medical staff and policymakers, which may lead to the one-sidedness of the research results.

*Cultural specificity*: The study’s findings are based on the Chinese sociocultural context, especially the participants’ strong demand for TCM-integrated palliative care, which limits the transferability of the research results to other cultural contexts (e.g., Western countries).

## 5 Conclusion

Based on the findings of this study, it is evident that end-of-life patients hold diverse expectations regarding hospice and palliative care services. It is imperative to fully respect patients’ decision-making rights and address their varying needs concerning care settings, service capabilities, and support systems. Every terminally ill individual deserves access to high-quality palliative care, enabling them to truly attain quality of life in their final days. Furthermore, there is an urgent need to develop a localized palliative care service model that ensures accessibility, practicality, and scalability across different regions and populations.

## Data Availability

The raw data supporting the conclusions of this article will be made available by the authors, without undue reservation.
